# Spontaneous Recession Repair after Orthodontic Treatment: Case Report with the Use of Digital Approach for Quantification of Soft Tissue Changes

**DOI:** 10.1155/2023/1831125

**Published:** 2023-06-24

**Authors:** Myroslav Goncharuk-Khomyn, Oleksandr Krasnokutskyy, Mykola Boichuk, Vitaliy Rusyn, Marharyta Hliudzyk-Shemota

**Affiliations:** ^1^Faculty of Dentistry, Uzhhorod National University, Uzhhorod, Ukraine; ^2^Faculty of Dentistry, I. Horbachevsky Ternopil National Medical University, Ternopil, Ukraine; ^3^Biological Faculty, Uzhhorod National University, Uzhhorod, Ukraine

## Abstract

The article presents a case of spontaneous recession repair in a male patient with Class II malocclusion, division 1, after orthodontic treatment with aligners. The difference in digital recession depth was measured before and at the end of treatment by means of automatic intraoral scans superimposition within adapted software while using “Cross section” and “Measuring” instruments. Digital analysis of intraoral scans obtained before and at the end of treatment has revealed that recessions within the area of teeth 1.5, 1.4, 1.3, 1.2, 1.1, 2.1, 2.2, 2.3, 2.4, and 2.5 have improved, and recession depth reduced by 0.73 ± 0.08 mm, 1.02 ± 0.09 mm, 1.86 ± 0.13 mm, 0.72 ± 0.09 mm, 0.73 ± 0.04 mm, 0.67 ± 0.06 mm, 0.66 ± 0.07 mm, 1.50 ± 0.12 mm, 1.10 ± 0.05 mm, and 0.45 ± 0.04 mm, appropriately. The present case report emphasizes that orthodontic correction of altered tooth position (angulation, inclination, and rotation) under certain clinical conditions may be considered as an effective method for soft tissue contour optimization in cases when pre-treatment tooth position could be interpreted as a causative factor or associated with diagnosed recession. The following outcomes could be related, but not limited to creeping attachment mechanism, bone-housing centering effects, optimization of occlusal load distribution with ruling out peak zones of strain accumulation, and mucogingival stress leveling. Due to the authors' knowledge, the present case report is the first one where the signs of spontaneous recession repair after orthodontic treatment were evidenced with the intraoral scans and quantified by the specifically implemented digital analysis approach.

## 1. Introduction

Gingival recession is one of the most prevalent pathological conditions of gingival margin among dental patients, which is associated with root surface exposure, tooth sensitivity, periodontal attachment loss, increased risk of tooth caries development, and compromised esthetic profile [1, 2]. Even though potential associations between gingival recessions and orthodontic treatment remain a debatable issue, some of the available evidences have demonstrated an increased risk of recession development among orthodontically treated subjects compared to untreated individuals [3, 4].

Meanwhile, anecdotic case reports available in the literature represent outcomes opposite to such mentioned above and are associated with the recessions' improvement after provided orthodontic treatment [5–9]. Some clinical cases also have shown spontaneous gingival recession closure after orthodontic treatment combined with some recession-oriented grafting procedures, but in presented reports, it was difficult to distinguish if the gingival margins improvements were caused by the periodontal surgery or foremost due to the orthodontic treatment itself [10]. Creeping attachment phenomenon was interpreted also as one of the possible reasons for recessions repair after isolated or combined orthodontic treatment [10].

Various parameters were proposed to quantify the recession characteristics following orthodontic and periodontal procedures [11–13]. Clinical crown height and gingival margin-papillae measurements were evidenced to be reliable quantitative references for recession changes assessment in clinical practice and for the research purposes [11]. Even though clinical measurements of recession parameters help to evaluate not only recession depth and width, but also their correspondence to potential clinical attachment loss, thickness of gingiva, and depth of the periodontal probing, it is quite a time-consuming procedure, which requires sufficient clinical experience and calibration to obtain reliable and reproductive results [11–13].

On the other hand, several previous studies have demonstrated the advantages of using digital intraoral scanning approach in terms of recession level registration, while pointing to the proper validity, reliability, and accuracy of such method [14–17]. Ongoing digitalization in dentistry is supported by the unique possibilities of innovative available techniques and equipment, which further could be implemented in various clinical settings and for different specific objectives. Nowadays, even automated analysis technique has been developed specifically for the gingival recession measurements with the use of obtained intraoral scans [18].

Based on the authors' knowledge, there is no available case report demonstrating spontaneous improvements of gingival recessions after solely orthodontic treatment and with evidences presented specifically with intraoral scans, but not only with clinically collected data and photodocumentation. Moreover, quantification of gingival margin changes with the use of digital analysis approach either after periodontal surgery or after orthodontic treatment remains a relevant issue both for the clinical practice and also of high scientific interest.

The objective of this study was to present the clinical case of spontaneous partial recession repair after orthodontic treatment evidenced by the intraoral scans and quantify associated positive gingival margin changes based on the adapted digital analysis.

## 2. Methods

Present case report followed CARE checklist and CARE guidelines, while also has been realized and presented in accordance with the ethical standards of Helsinki Declaration of 1975, as revised in 2002. The patient agreed on his intraoral scans being published for demonstration reasons, while he refused X-ray examination data and clinical photos being published for the same purposes, and considering these facts, patient has signed the adapted form of informed consent.

The intraoral scanning procedure was performed with 3Shape TRIOS 3 wired scanner (3Shape, Copenhagen, Denmark). Orthopantomogram (OPG) was obtained on PROMAX 3D Classic unit (Planmeca OY, Helsinki, Finland). Parameter of recession depth (RecDep) defined as the distance between cementoenamel junction level and the position of free gingival margin in the projection of recession was measured both clinically and with the use of obtained intraoral scans. Clinical assessment of recession depth (RecDepClin) has been accomplished with the use of the North Carolina periodontal probe in millimeters (mm), while digital evaluation of recession depth (RecDepDig) was performed in 3Shape adapted software 3Shape Clear Aligners Studio (3Shape, Kyiv, Ukraine) [13]. The difference in clinical recession depth before and by the end of treatment (∆RecDepClin) has been calculated as the mathematical difference of RecDepClin parameter before and by the end of treatment, while the difference in digital recession depth before and by the end of treatment (∆RecDepDig) was measured after automatic intraoral scans superimposition within 3Shape adapted software using “Cross section” and “Measuring” instruments.

The parameter of mean root coverage deficiency (MRCD) was estimated based on the received digital scans by the end of orthodontic treatment as a difference between potential full soft tissue coverage to cemento-enamel junction (CEJ) level as a reference line and resulted soft tissue coverage of the root surface in the area of recession, represented in percentage (%) [13].

Personal patient's satisfaction with the esthetic appearance of dentogingival profile was measured according to visual analogue scale (VAS) in the range from 0 points (full unsatisfaction with dentogingival profile appearance) to 10 points (full satisfaction with dentogingival profile appearance) [13].

All the clinical measurements were conducted by the same dental specialist, while all the digital measurements were provided by two authors of the present manuscript, who underwent preceding calibration for comparative non-randomized clinical trial, which was also dedicated to the digital and clinical evaluation of recession parameters [13].

## 3. Statistical Analysis

Differences between recession depth changes, registered clinically and digitally, were statistically affirmed only under condition of *p* < 0.05 (significance level of 0.95). Paired Student's *t*-test has been used to compare the clinically and digitally registered changes of recession depth. Considering the care report design of the present study, no sample size calculations were provided specifically for this research. Inter- and intra-rater agreement levels have been assessed by Cohen's kappa coefficient. Data accumulation with its further stratification and tabulation as well as necessary inferential statistical processing was held within Microsoft Excel software version 16.0 (Microsoft Office 2019, Microsoft Corporation India Pvt. Ltd., New Delhi, India).

## 4. Case Report

Male 40-year-old patient has been referred to dental clinic owned by the first author (3Dplus Dental Clinic, Cherkasy, Ukraine) with the main complaint of being non-satisfied with his present esthetic appearance of maxillary teeth. During clinical examination, it was revealed that the patient had Class II malocclusion, division 1, since mesiobuccal cusps of maxillary first molars were occluding anteriorly to the buccal grooves of mandibular first molars with proclined maxillary central incisors.

**Figure 1:**
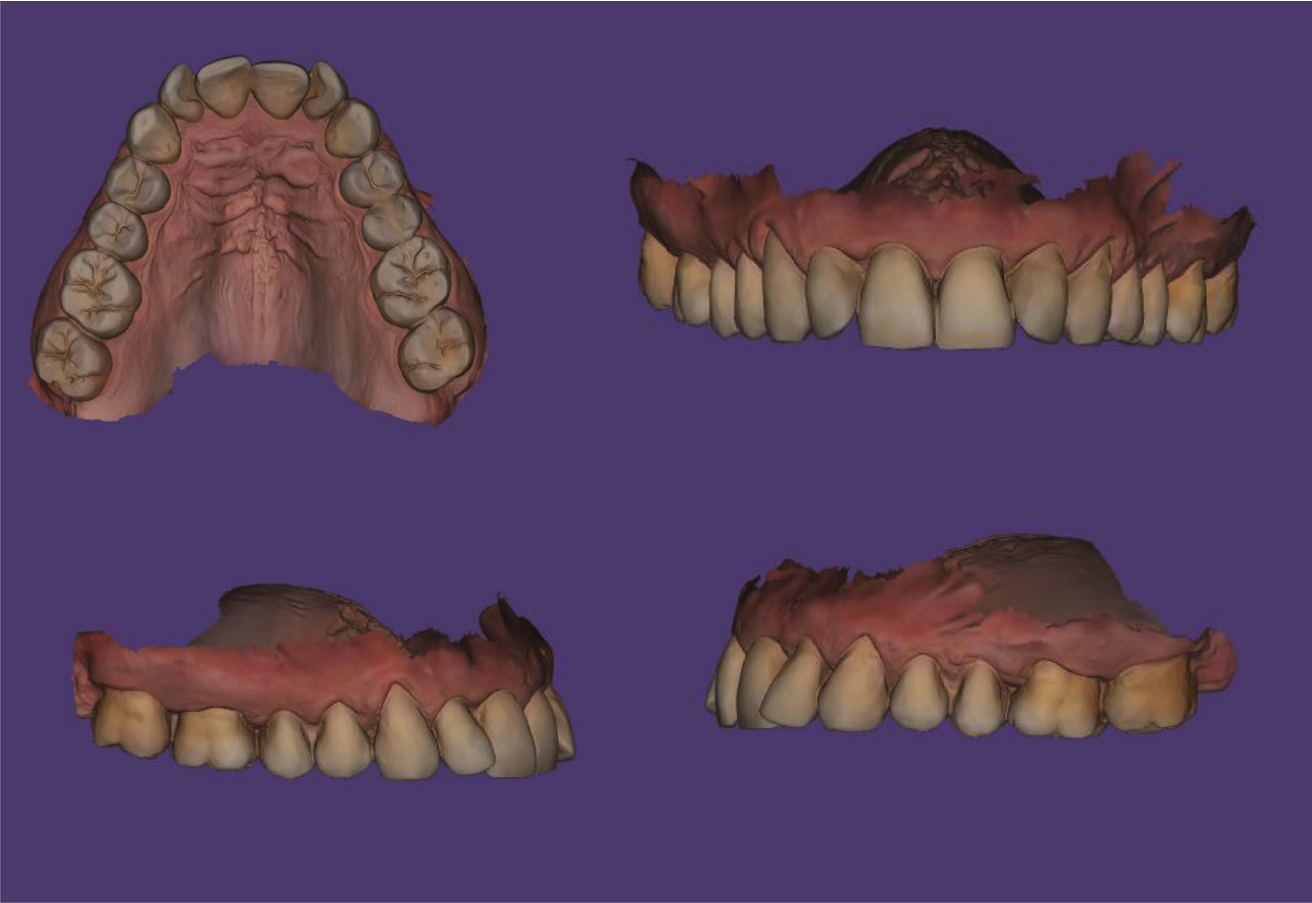
Patient's intraoral maxilla scans before the orthodontic treatment.

**Figure 2:**
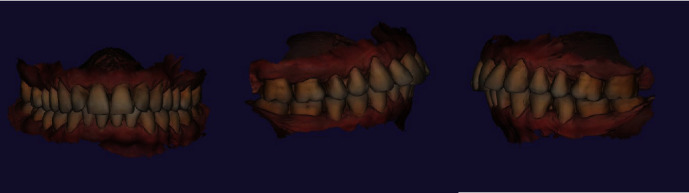
Patient's intraoral bite scans before the orthodontic treatment.

Also, teeth crowding was observed in the frontal region of the maxilla, while teeth 1.2 and 2.2 presented signs of rotations. Recessions categorized as I class by Miller were noted in the area of teeth 1.5, 1.4, 1.3, 1.2, 1.1, 2.1, 2.2, 2.3, 2.4, and 2.5, which were the part of patient's complaint regarding esthetic profile compromise (Figures 1 and 2).

Teeth 1.7, 1.6, 2.6, and 2.7 also revealed signs of I class recessions, but the patient did not recognize those as a part of the clinically important problem even after visualizing them on the obtained picture presenting current clinical situation. OPG (Planmeca OY, Helsinki, Finland) revealed the absence of the third molars both at maxilla and mandible, and during clinical examination, no critical differences between sizes of the maxilla and the mandible were noted. Patient was proposed to undergo a partial orthodontic correction with aligners system to optimize occlusal interrelations between maxillary and mandibular dentition as a pre-prosthetic stage of rehabilitation.

The prosthetic treatment plan assumed final correction of 1.6–2.6 teeth contours, positions, and occlusion alignments with the use of full-ceramic crowns after orthodontic intervention. If the patient is satisfied with obtained clinical results, further prosthetic treatment of lower dentition will be started. The patient agreed on the proposed treatment plan and signed appropriate consent form.

Previously, the patient has not undergone any kind of orthodontic treatment. The orthodontic phase of the complex treatment plan included the use of aligners (Clear Aligners, Smile Correct, Midway Dental Laboratory, Shenzhen, China). Before any interventions, intraoral scans of mandibular dentition, maxillary dentition, and occlusal bite were obtained and sent to the dental laboratory for further aligners treatment planning. The developed treatment plan consists of the use of 11 successive aligners on the maxilla as well as 11 aligners on the mandible. The aligner attachments were fixed on teeth 1.5, 1.4, 1.3, 1.2, 1.1, 2.1, 2.2, 2.3, 2.4, and 2.5 maxillary, while orthodontic separation of 0.3 mm was provided in the interproximal areas of teeth 1.4–1.5, 1.5–1.6, 2.4–2.3, 2.5–2.4, and 2.6–2.5. On the mandible, aligner attachments were fixed on teeth 3.5, 3.4, 3.3, 3.2, 3.1, 4.1, 4.2, 4.3, 4.4, and 4.5, while orthodontic separation of 0.3 mm was provided in the interproximal areas of teeth 3.3–3.4, 3.4–3.5, 3.5–3.6, 4.5–4.4, and 4.6–4.5, and orthodontic separation of 0.2 mm was provided in the interproximal area of teeth 3.1–3.2, 4.1–3.1, and 4.2–4.1.

**Figure 3:**
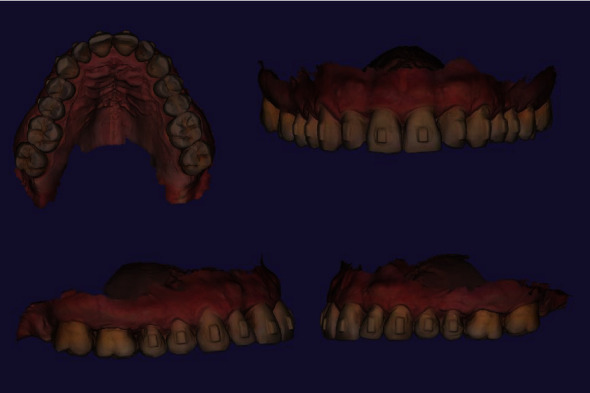
Patient's intraoral maxilla scans by the end of orthodontic treatment.

**Figure 4:**
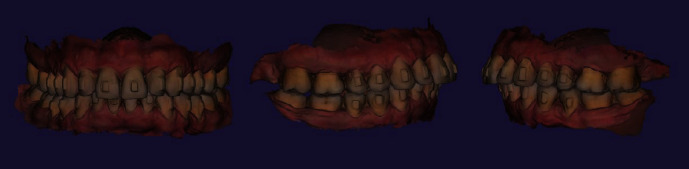
Patient's intraoral bite scans by the end of orthodontic treatment.

Orthodontic treatment has been started in March of 2021 and went on till July of 2022. At the last visit before attachment separation, repetitive intraoral scans were obtained including mandibular dentition, maxillary dentition, and occlusal bite. Both clinical examination and provided digital analysis of obtained intraoral scans revealed improvement of gingival margins at the area of teeth 1.5, 1.4, 1.3, 1.2, 1.1, 2.1, 2.2, 2.3, 2.4, and 2.5 (Figures 3 and 4). By the end of orthodontic treatment, no inflammatory-associated gingival changes or pathological overgrowth of gingiva were noted during clinical examination before attachment separation.

All the clinical improvements after orthodontic correction were demonstrated to the patient within the intraoral scanner-associated software with dynamic visualization of the partial recession repair, which took place during the treatment. Patient has been fully satisfied with the obtained results and overall improvements of the final maxillary teeth esthetic appearance. He refused to undergo any further treatment as long as obtained visual profile of the maxillary dentition along with achieved pink and white esthetics has fully satisfied his personal expectations.

Patient has been fully informed about the possibility of providing further prosthetic treatment in the nearest or remote future, and for now, he adheres to the scheduled periodical follow-up visits for regular check-ups.

Three months after orthodontic treatment, no inflammatory-associated changes of gingiva have been noted, and no pathological overgrowth or reduction of gingiva has been registered; the gingival margin was stable and close to the same measurements registered by the end of orthodontic treatment.

## 5. Results

Changes in teeth positions were calculated automatically after superimposing intraoral scans obtained before and by the end of treatment in the adapted software. The specific parameters of teeth rotation as well as changes in angulation and inclination parameters are presented in Table 1.

Provided orthodontic treatment provoked corpus dislocation of tooth 2.1 to the side and forward on 0.1 mm, and also its intrusion on −0.1 mm. The signs of extrusion were also noted at the teeth 4.4 (0.2 mm), 4.3 (0.3 mm), 4.2 (0.2 mm), 4.1 (0.2 mm), 3.2 (0.2 mm), 3.3 (0.3 mm), and 3.4 (0.2 mm).

**Figure 5:**
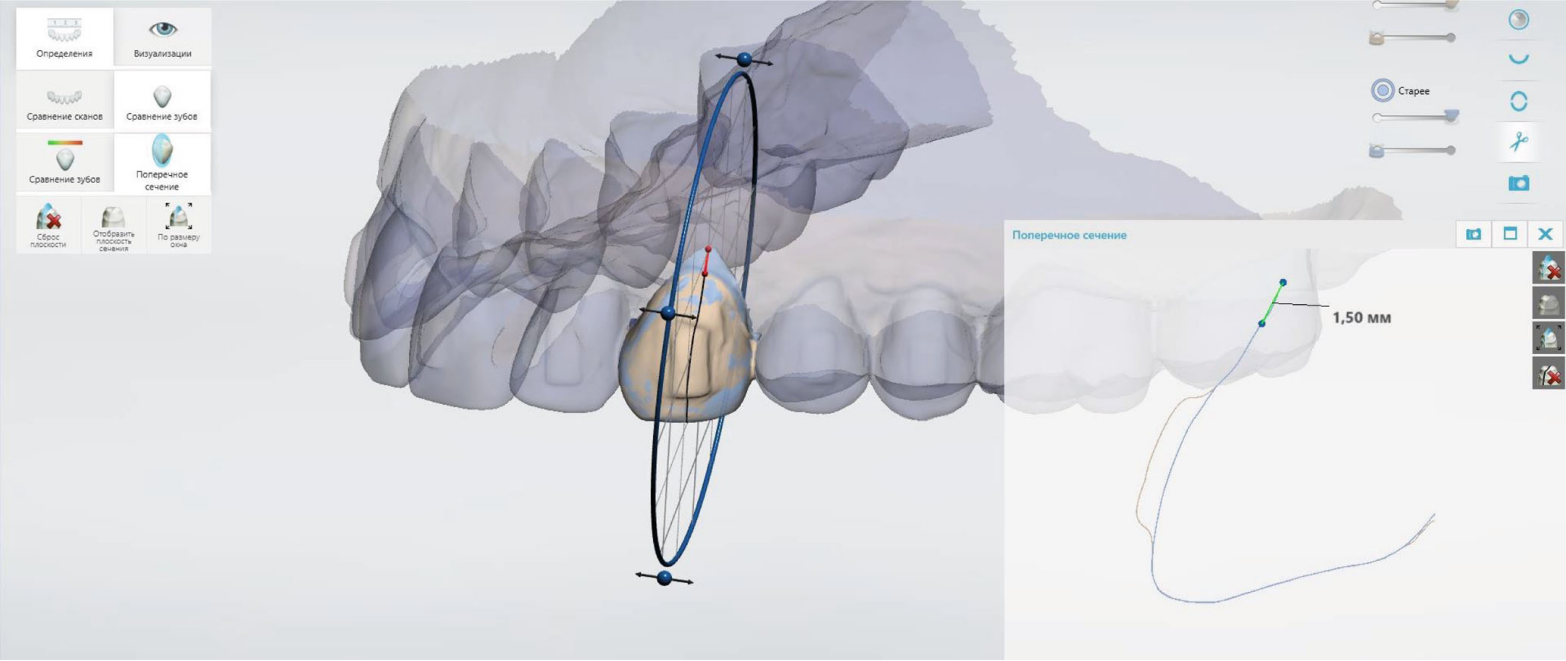
Demonstration of recession repair by 1.5 mm at the area of tooth 2.3 using the superimposition of intraoral scans as well as “Cross section” and “Measuring” tools in adapted software.

**Figure 6:**
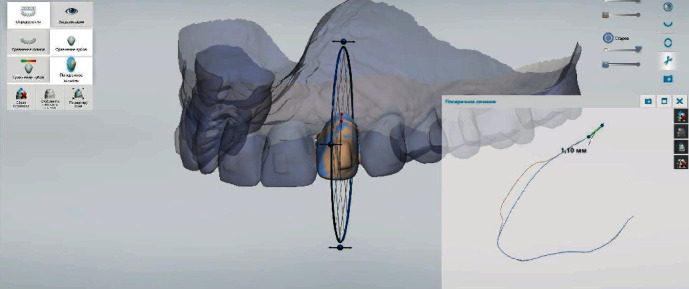
Demonstration of recession repair by 1.10 mm at the area of tooth 2.2 using the superimposition of intraoral scans as well as “Cross section” and “Measuring” tools in adapted software.

**Figure 7:**
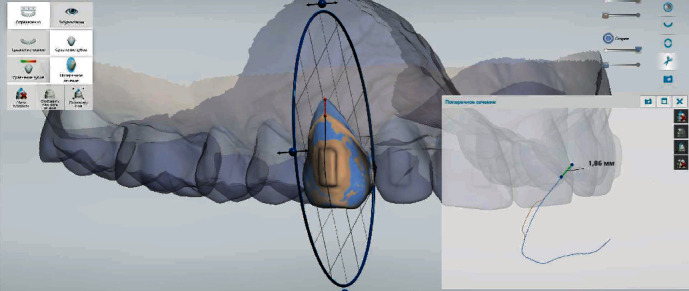
Demonstration of recession repair by 1.86 mm at the area of tooth 1.3 using the superimposition of intraoral scans as well as “Cross section” and “Measuring” tools in adapted software.

The digital analysis of intraoral scans obtained before and by the end of treatment revealed that recessions at the area of teeth 1.5, 1.4, 1.3, 1.2, 1.1, 2.1, 2.2, 2.3, 2.4, and 2.5 have improved, and recession depth reduced by 0.73 ± 0.08 mm, 1.02 ± 0.09 mm, 1.86 ± 0.13 mm, 0.72 ± 0.09 mm, 0.73 ± 0.04 mm, 0.67 ± 0.06 mm, 0.66 ± 0.07 mm, 1.50 ± 0.12 mm, 1.10 ± 0.05 mm, and 0.45 ± 0.04 mm, appropriately (Figures 5, 6, and 7).

By the end of orthodontic treatment phase, the average root coverage deficiency ranged from 12.16 ± 3.24% to 17.22 ± 3.73%, while not being statistically different at the areas of analyzed teeth (Table 2).

Both clinical method and digital analysis method have shown identical results in terms of changes in recession depth with no difference registered between methods (*p* > 0.05).

Intra-rater reliability of recession depth clinical measurements reached 0.80, while digital measurements of recession depth reached 0.95 level of intra-rated reliability. Inter-rater reliability in terms of digitally registered differences in recession depth before and by the end of treatment reached Cohen's kappa value of 0.91.

**Figure 8:**
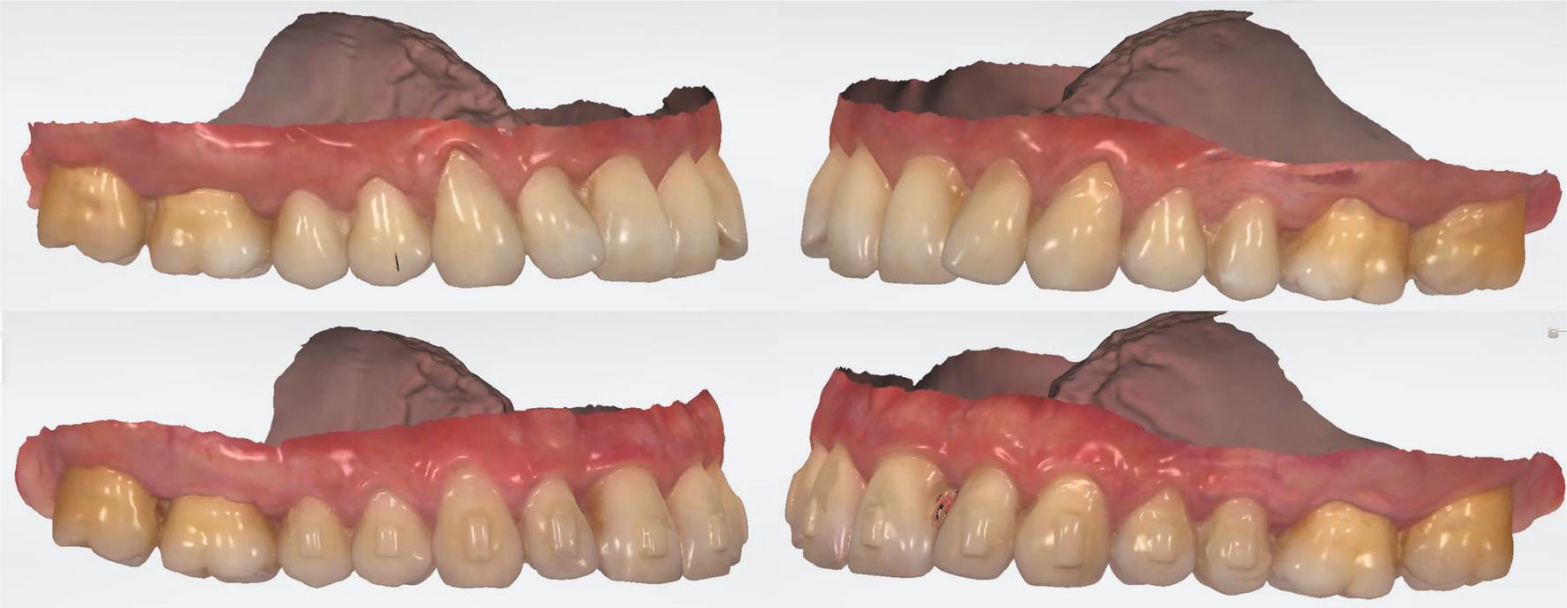
Intraoral scans of maxilla before (upper figures) and by the end (lower figures) of orthodontic treatment. Signs of recession repair are visible at the area of frontal teeth (1.5–2.5).

Before treatment, the patient assessed the esthetic dentogingival appearance of his frontal maxillary area as equal to 5 VAS points, while by the end of orthodontic correction, his assessment reached 9 points (Figure 8).

## 6. Discussion

The recent systematic review has shown that the factual amount of recessions is not characterized by significant differences among patients who underwent orthodontic treatment and those who did not receive any kind of orthodontic intervention due to normal occlusal pattern [3]. Tepedino et al. highlighted a lack of strong evidences regarding statistical and clinical effects of orthodontic treatment on gingival recession development [19]. Moreover, even recession areas grafted by subepithelial connective tissue before orthodontic interventions were characterized by stability throughout the whole orthodontic treatment course [20]. On the other hand, even though orthodontic treatment accordingly to the available literature data cannot be categorized as the major risk factor for gingival recessions [21], some increase in recession prevalence was noted not by the end of active phase, but during further remote retention period after orthodontic corrections [22]. Also, increase of tooth inclination labially during the orthodontic treatment tends to be related to gingival margin reduction (0.2 mm per 1°) [23]. The present case report has demonstrated the opposite effect of orthodontic intervention: correction of maxillary teeth position with aligners supported spontaneous recession repair after 16 months of treatment.

In the present case report, it was possible to provide prosthetic treatment without any orthodontic correction and change in patient's dentogingival appearance with the newly formed esthetic profile of crowns and bridges. Nevertheless, several previous studies demonstrated a positive effect of orthodontic correction on the gingival contour changes, while also on the optimization of occlusal relationship [5–10]. Considering available evidences, it was chosen to provide pre-prosthetic orthodontic treatment to improve complex treatment outcomes [5–10].

Several studies previously have described very similar results of recession repair after provided orthodontic treatment. Analysis of 12 clinical cases of orthodontic adult therapy performed by Laursen et al. reported an average recession depth decrease by 23%, recession width decrease by 38%, and initial recession area reduction by 63% due to the “root centering within bone housing effect” obtained after orthodontic correction [5]. Antanavičienė et al. reported about improvement of 58.7% recession cases, while 36% of the analyzed recessions were stable, and 5.3% demonstrated progression after performed orthodontic interventions [1]. The case report of de Figueiredo et al. demonstrated significant gingival recession and bone dehiscence reduction after the usage of clear aligner system, which helped to move the tooth root into the proper position within the alveolus [6]. Analogical positive recession repair outcomes were also reported during orthodontic treatment of anterior cross-bite [7] and Class II (division 1) malocclusion [8].

It should be stated that not all cases of malocclusion associated with recessions demonstrated analogical trends of gingival recessions improvements after orthodontic treatment. Class III malocclusion and open-bite conditions were found to restrain gingival recession improvement after orthodontic therapy [1]. Ji et al. reported that open-bite and infraversion patients who underwent orthodontic treatment were characterized by statistically higher prevalence of gingival recession after the treatment completion [24]. Meanwhile, the influence of such factors as gingival biotype, urgency of tooth extraction, and tooth inclination on the risk of gingival recession development during or after the orthodontic treatment should not be underestimated [23–25]. The following pre-treatment factors as the height of keratinized gingiva and the width of mandibular symphysis as well as post-treatment intercanine width have been categorized as predictors of gingival recession development during different orthodontic therapy approaches [26].

The creeping attachment was categorized as one of the mechanisms potentially related to gingival recession improvements after orthodontic treatment combined with periodontal surgery [10]. Phenomenon of creeping attachment mostly has been interpreted as a remote effect of specifically periodontal surgeries for recession treatment after the end of “bridging” period and graft maturation. Nevertheless, in the early publication of Machado et al., partial repair of the gingival recession has been obtained after orthodontic correction of the mandibular right central incisor with pronounced labial torque, which, as authors believed, is related to creeping attachment [9]. Even though this case report has been dated 10 years ago, it is still represented a great interest since two periodontal surgeries performed before orthodontic treatment and targeted to recession closure at the projection of problematic incisor had failed [9].

In the previous studies, verification of positive recession changes after provided orthodontic treatment has been performed either clinically, based on obtained intraoral photos [5], or during analysis of plaster models (dental casts) [1]. In the present case report, we have used a digital analysis approach, a version of which authors have previously implemented to quantify recession changes after using a xenogeneic matrix, which has been called a digital soft tissue design [13].

Nevertheless, several more successful approaches were described in the literature in terms of digital evaluation of gingival recession [16]. Moreover, the digital approach used for the measurements of gingival recession was found to be more reproducible and valid compared to the conventional clinical examination [14–17]. In the present case report, we could not find any statistical differences between changes in the gingival recession parameters measured by means of digital analysis or conventional clinical method.

The use of an intraoral scanner for soft tissue change analysis, which is not fully free of lapses but optimizes the approach for dynamic monitoring, is able to facilitate the data accumulation for further in-detail comparison and simplifies the measuring process itself within the required clinical settings when the operator should be familiar with the basics of intraoral scanning and graphic images processing [16, 18].

A spontaneous repair of gingival recession after performed orthodontic treatment has not been studied systematically so far, since only several case reports and case series are available in this matter. Nevertheless, more attention should be paid to this aspect, since it opens potential perspectives of applying an orthodontic therapy, among other purposes, with the aim of soft tissue contour optimization before prosthetic phase of rehabilitation. The latter could be implemented into the complex prosthetic treatment protocol for patients with various forms of dentoalveolar abnormalities, or at least it could minimize the number and volume of required surgical interventions to restore proper soft tissue coverage above the exposed root surface. Optimization of the orthodontic treatment itself also may be provided with the usage of the different medications and compounds, which are able to induce positive soft tissue changes and minimize the risk of potential complication occurrence [27–30].

The limitations of the present case report are associated with no cone beam computed tomography (CBCT) examination provided for the patient before and after orthodontic treatment, since orthodontic planning was case-dependent and included only orthopantomography as an initial method of diagnostics before the treatment initiation. The availability of CBCT data would support the possibilities for analysis of tooth root proclination changes and how they corresponded to the bone envelope margins before and after orthodontic correction of teeth position. Visually, it was noted that a labial inclination of teeth with recessions has been reduced after orthodontic treatment, but again X-ray evidence of those changes would be more objective. Previously, it was suggested that an increase of tooth proclination had been associated with a higher occurrence of gingival recession, while retroclination itself had demonstrated much lesser connection with gingival margin apical displacement [23]. Another limitation of the study has been related to the fact that all clinical measurements were provided only by one dental clinician. Nevertheless, it should be noted that this clinician was previously calibrated due to the participation in non-randomized clinical research, while also the objective of this study was to demonstrate the possibilities of using the specific digital intraoral scans for evidencing gingival recession repair. The analysis of obtained digital scans was provided by two clinicians, while the results obtained through digital analysis were characterized by a high level of intra- and inter-rater reliability.

## 7. Conclusion

Considering the findings of present case report and the data available in the literature, it may be assumed that orthodontic correction of altered tooth position (angulation, inclination, and rotation) under certain clinical settings may be considered as an effective method for soft tissue contour optimization in cases when the pre-treatment tooth position could be interpreted as a causative factor or associated with diagnosed recession. Such outcomes could be related, but not limited to creeping attachment mechanism, bone-housing centering effects, optimization of occlusal load distribution with ruling out peak zones of stress accumulation, and mucogingival stress leveling. Due to the authors' knowledge, the present case report is the first one, where signs of a spontaneous recession repair after orthodontic treatment were evidenced with the intraoral scans and quantified by the specifically implemented digital analysis approach.

## Figures and Tables

**Table 1:** Changes in teeth rotation, angulation, and inclination parameters after orthodontic treatment with aligners.

Tooth	Rotation changes (°)	Angulation changes (°)	Inclination changes (°)
1.5	0	−1.6	2.4
1.4	0	−2.1	1.9
1.3	0	−2.1	3.1
1.2	−22.1	−2.7	1.0
1.1	−2.5	0	3.6
2.1	3.4	0.9	5.0
2.2	−18.7	−1.7	−0.2
2.3	−8.7	−2.3	1.6
2.4	0	−2.1	2.0
2.5	0	−1.0	1.1
3.5	0	0	−1.5
3.4	0	−1.3	3.2
3.3	−5.8	−3.2	0.1
3.2	26.6	−0.5	−2.1
3.1	4.5	−0.2	−0.5
4.1	−3.4	−2.2	−2.9
4.2	10.5	−3.4	−0.7
4.3	0	−2.1	2.2
4.4	−3.9	−2.1	2.8
4.5	0	−1.2	2.6

**Table 2:** Changes in recession depth before and by the end of treatment with aligners (registered clinically and based on obtained intraoral scans).

Tooth	∆RecDepClin	∆RecDepDig	*p*-value	MRCD (%)
1.5	0.67 ± 0.14	0.73 ± 0.08	>0.05	13.18 ± 3.61
1.4	0.98 ± 0.17	1.02 ± 0.09	>0.05	15.45 ± 4.53
1.3	1.70 ± 0.22	1.86 ± 0.13	>0.05	15.79 ± 3.27
1.2	0.65 ± 0.15	0.72 ± 0.09	>0.05	15.22 ± 2.33
1.1	0.69 ± 0.16	0.73 ± 0.04	>0.05	12.16 ± 3.24
2.1	0.63 ± 0.13	0.67 ± 0.06	>0.05	17.53 ± 4.18
2.2	0.65 ± 0.14	0.66 ± 0.07	>0.05	17.22 ± 3.73
2.3	1.44 ± 0.20	1.50 ± 0.12	>0.05	16.34 ± 4.85
2.4	1.02 ± 0.12	1.10 ± 0.05	>0.05	17.21 ± 2.73
2.5	0.40 ± 0.08	0.45 ± 0.04	>0.05	14.37 ± 3.09

∆RecDepClin: difference in clinical recession depth before and by the end of treatment; ∆RecDepDig: difference in digital recession depth before and by the end of treatment; MRCD: mean root coverage deficiency.

## Data Availability

Data supporting this research article are available from the corresponding author or first author on reasonable request.
